# The management of a breast unit during the COVID-19 emergency: a local experience

**DOI:** 10.2217/fon-2021-0243

**Published:** 2021-10-21

**Authors:** Daniele Ugo Tari, Aldo Santarsiere, Fabiola Palermo, Caterina Desireè Morelli, Fabio Pinto

**Affiliations:** ^1^Department of Diagnostic Senology, DS12, Caserta LHA, 81100, Caserta (CE), Italy; ^2^Department of Pathological Anatomy A. di Tuoro, Caserta LHA, 81031, Aversa (CE), Italy; ^3^Department of Radiology, A. Guerriero Hospital, Caserta LHA, 81025, Marcianise (CE), Italy

**Keywords:** breast neoplasms, clinical practice patterns, COVID-19, pandemics, screening

## Abstract

**Introduction:** Since breast imaging requires very close contact with patients, a protocol is needed to perform safe daily screening activities during the COVID-19 pandemic. **Materials and methods:** Patients were triaged and separated into three different clinical scenarios by performing a telephone questionnaire before each diagnostic exam or a nasopharyngeal swab before every recovery. Specific procedures for each scenario are described. **Results:** From July to October 2020, 994 exams were performed. A total of 16 cancers and 7 suspected COVID-19 patients were identified. No medical were was infected. **Conclusion:** This protocol is an example of the practical use of guidelines applied to a breast unit to assist specialists in preventing COVID-19 infection and optimizing resources for breast cancer diagnosis.

## Lay abstract

On March 11th, 2020, the WHO officially declared the COVID-19 infection pandemic. Since breast cancer represents the most frequent cancer in women of all ages, and breast imaging examinations require very close contact with patients, a protocol was designed to optimize the management of patients and healthcare workers, performing strict COVID-19 screening and avoiding any impairment of survival of patients with breast cancer. Patients were separated into three different clinical scenarios (non-COVID-19 patients, suspected COVID-19 patients and confirmed COVID-19 patients) by performing a telephone questionnaire before each diagnostic exam or a nasopharyngeal swab before every recovery. Specific procedures for each scenario are described. Confirmed or suspected patients are rescheduled if not urgent. From July to October 2020, 994 exams were performed. A total of 16 cancers and 7 suspected COVID-19 patients were identified. No medical staff were infected. This study demonstrates efficacy in terms of continuity in the provision of an essential level of care in a breast cancer screening and ambulatory setting, providing an example of the practical use of guidelines applied to a breast unit, to assist specialists in preventing COVID-19 infection and optimizing resources for breast cancer diagnosis.

On January 30th, 2020, the WHO officially declared the coronavirus disease 2019 (COVID-19; SARS-CoV-2) epidemic as a public health emergency, and, on March 11th, officially defined the rapid universal spread of infection as a pandemic [1,2]. On June 30th, WHO data reported 17,106,007 confirmed cases worldwide since the start of the outbreak and 668,910 deaths. On the same date, in Italy, 247,158 cases had been confirmed, including 35,132 deaths reported to the WHO [2,3]. The predominant virus transmission mechanism is by droplet contact. Other mechanisms of infection include contact with infected surfaces touched by people who, without correct hands disinfection, then touch their own mouth, nose or eyes [1]. The risk of being infected also depends on physical proximity [4]. People are often infectious 2–3 days before they exhibit symptoms, so the proportion of presymptomatic transmission ranges from 48% to 62% [5]. Spread from asymptomatic carriers is estimated at 25% [6]. Thus, solely screening for symptoms does not provide protection for all people [6].

There is no chance of physical distancing when performing breast imaging, such as mammography or ultrasound (US), or breast intervention procedures, such as US-guided fine-needle agoaspiration cytology (US-FNAC), US-guided core needle biopsy (US-CNB) and vacuum-assisted breast biopsy (VABB), so that a radiologist or a radiographer works within 20–30 cm of the patient's face. During the outbreak, breast screening activities were interrupted, as resources were routed to COVID-19 care; accordingly, only cancer emergency-related situations remained ongoing. More recently, from July 1st, screening activities have resumed. Nevertheless, as strategies to prevent the spread of the infection are mandatory, based upon our experience at the Breast Unit of the Caserta Local Health Authority, focusing on patients undergoing their first diagnostic exam at the Department of Diagnostic Senology, District 12, subsequently sent to the Department of Breast Surgery, A. Guerriero Hospital, a protocol was developed to optimize the management of patients and medical staff, performing strict COVID-19 screening and avoiding any impairment of the survival of patients with breast cancer. The aim of this study is to present this protocol, which was focused on patient management and schedules. International guidelines were followed for the protection of patients and staff.

## Materials & methods

Literature and guidelines have identified four phases of SARS-CoV-2 infection, depending on the country's COVID-19 status [7]:

- Phase 1 – semiurgent setting: severe disease with no community spread. Hospital resources (ICU beds, ventilator capacity and medical supplies) are ample. Operational changes are focused on resource rationalization and preparedness.
- Phase 2 – urgent setting: severe disease with contained community spread. Large but controlled number of COVID-19-infected patients with no overload of hospital resources. Operational changes are focused on the progressive reduction of case numbers.
- Phase 3 – lockdown: severe disease with uncontained community spread (spike of cases) even with containment procedures in place. Hospital resources are stressed but sufficient, with significant redirection to COVID-19 care.
- Phase 4 – uncontrolled pandemic: severe disease with uncontrolled spread. Resources are exhausted even if fully directed to COVID-19 care.

According to the above classification, following a period of lockdown and a gradual easing of containment measures, on June 15th, Italy went back to a phase 1, semiurgent setting, so that screening activities resumed on July 1st. The SIRM Italian College of Breast Radiologists [8] promoted guidelines to protect both patients and healthcare workers (HCWs; including radiologists, radiographers, nurses and reception staff) against infection or disease spread during breast imaging procedures. In an effort to optimize human and technological resources, and based upon a clinical practice point of view, three kinds of scenarios can be identified:

1. Non-COVID-19 patient = first scenario.
2. Suspected COVID-19 patient = second scenario.
3. Confirmed COVID-19 patient = third scenario.

**Figure 1.**
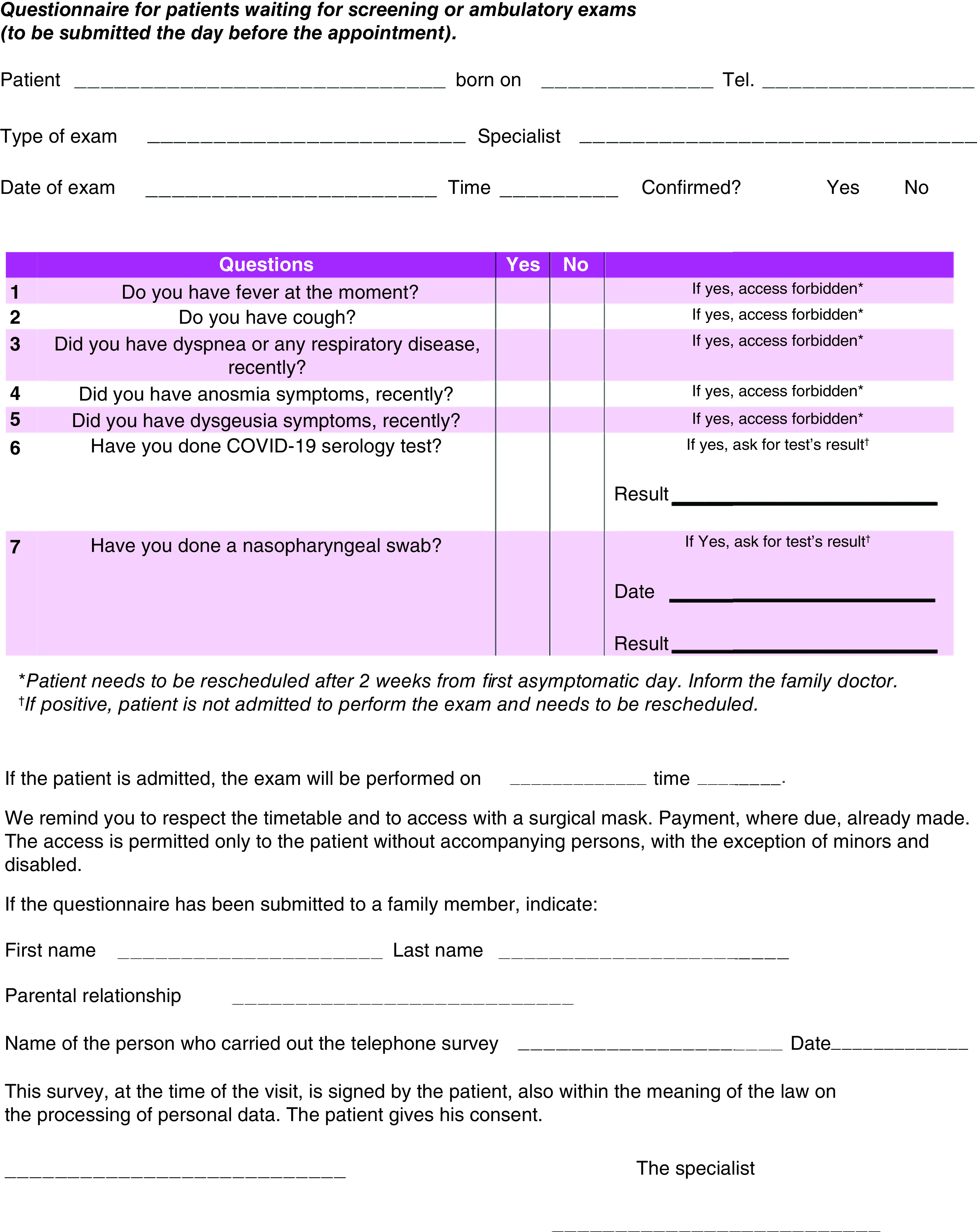
SARS-CoV-2 infection telephone questionnaire. If any answer is positive, the patient will not be admitted to the radiological exam and rescheduled after a complete health evaluation is made by his/her general practitioner doctor.

A non-COVID-19 patient is defined using a reverse transcriptase-polymerase chain reaction (RT-PCR) test, with a negative result. Since laboratory tests are not still validated for screening purposes, and considering the high number of asymptomatic or paucisymptomatic cases, HCWs must treat all patients as if they were infected [9]. Nevertheless, since the most common symptoms of the disease are fever, coughing and shortness of breath, a SARS-CoV-2 infection telephone questionnaire was created (Figure 1) to identify both positive and clinically suspected COVID-19 patients who must be postponed and rescheduled after two weeks from the first asymptomatic day. The first asymptomatic day was established by a complete health evaluation made by the general practitioner doctor (GP). The above statements followed the recommendations of national legislation ISS COVID-19 n. 1/2020 and its revisions, properly adapted to our local requirements [10].

For patients admitted to procedures, hand sanitization and body temperature checks are performed before entering the department; then, the medical and paramedical staff must follow specific procedures according to the identified clinical scenario. The time-lapse between the exams is 20 min, taking into account patient accommodation, exam time, accurate machine and room disinfection, and air ventilation (at least 10 min are needed).

At the Department of Diagnostic Senology, both screening and ambulatory outpatients are routinely welcome. The first group includes women aged 50–69 or >40 years with a high risk for breast cancer (familiarity, genetics, breast density), collected through the Sani.ARP web portal [11] who present for mammography as the first-level exam. Ultrasound represents a second-level exam, usually to clarify mammography or digital breast tomosynthesis (DBT) findings. US can outperform mammography, particularly regarding younger patients with dense breasts.

The second group includes women of all ages who need a breast imaging exam (mammography or US) for symptoms, oncologic follow-up or extra-screening periodic evaluation. According to our Diagnostic-Therapeutic Care Pathway (DTCP) [12], a DBT in double projection (medio-lateral-oblique and cranio-caudal) with synthetic 2D view reconstructions is performed. Screening exams are validated in double-blind reading by two radiologists; ambulatory exams are validated in single reading by a dedicated radiologist.

Examinations that are more complex are scheduled on a single day, always following full specific safety procedures. In particular, a whole daily session per week is dedicated to breast interventional radiology, including FNAC, CNB and VABB. US-guided interventional procedures (FNAC and CNB) are routinely performed with lower costs than VABB. Furthermore, since these procedures have a similar risk to performing US examinations as standalone, only patients who underwent these exams were triaged. Patients recruited to undergo a VABB procedure, instead, received an RT-PCR test at the department two days before the exam. If negative, she/he would be admitted to perform the biopsy and discharged on the same day the procedure was completed. If the patient should undergo breast surgery, a second RT-PCR test was performed at recovery, and the patient is discharged as soon as she/he is clinically stable, usually two days after surgery. To follow-up, a dedicated well-trained breast care nurse or radiographer (the case manager) called the patient at home, on the first and third day after discharge to verify her/his clinical status.

### Methods at the department of diagnostic senology

**Figure 2.**
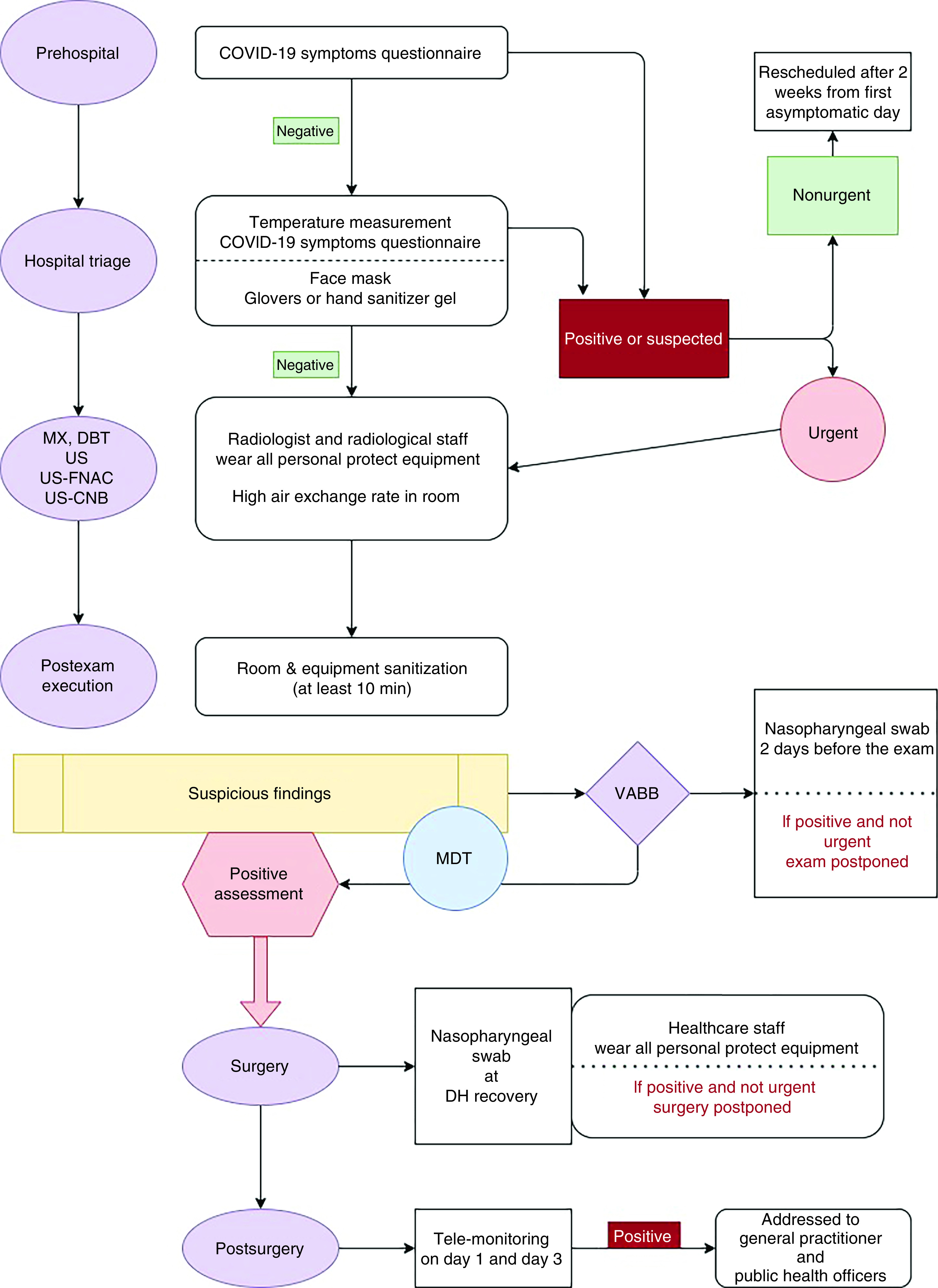
Flowchart outlining prehospitalization, inhospital and posthospital procedures. CNB: Core-needle biopsy; DBT: Digital breast tomosynthesis; FNAC: Fine-needle agoaspiration cytology; MDT: Multidisciplinary team meeting; MX: Mammography; US: Ultrasound; VABB: Vacuum-assisted breast biopsy.

According to the recommendations of national legislation ISS COVID-19 n. 1/2020 [10] and WHO recommendations released on February 2020 [2], properly adapted to our local requirements and as shown in Figure 2, all patients were subjected to a telephone triage the day before the radiologic exam by a dedicated breast radiographer. The pool of questions, shown in Figure 1, is asked again before the exam is performed, and finally evaluated by the radiologist. Body temperature measurement is performed for each patient before entering the hospital. Each appointment is scheduled every 20 min, to allow the exam execution (DBT plus 2D synthetic reconstructions), time for additional imaging (including magnification views or spot views) and time for equipment disinfection and air ventilation (at least 10 min). No more than two patients are allowed in the waiting room at the same time. No accompanying caregiver is allowed to stay, except for people with mental or physical disabilities requiring assistance. Two different doors are designated for entrance and for exit, respectively; the patient will not pass from through the same corridor twice and will not encounter the following patient. To avoid infection by SARS-CoV-2 for all medical and paramedical staff and for patients, additional procedures must be adopted, depending on different clinical scenarios:

- **First scenario – non-COVID-19 patient:** while waiting for a radiological procedure, all patients wear surgical masks and keep distance from other people (1 m). No other people, included accompanying people, are allowed to stay in the waiting room. The healthcare staff should wear surgical masks, avoids direct contact with the patient's oral and respiratory secretions, wear goggles or face shields and wear gloves and wash hands before wearing gloves and after removing them. A surgical cap and shoe covers are recommended. The ultrasound probe should be covered with a dedicated cap and disinfected after every procedure [9,13,14]. After each radiological procedure, the room and the radiological equipment must be cleaned and disinfected with chloro-derivate solutions and the room should be open for appropriate ventilation (>25 cycles/h) [9,15,16].
- **Second scenario – suspected COVID-19 patient (infection under investigation, patient waiting for swab results):** taking into account the highly contagious nature of SARS-CoV-2, these patients are not permitted to undergo breast imaging examinations until a negative result on the RT-PCR test is obtained, unless the needed exam is urgent (breast emergencies and urgent cancer included, locally advanced breast cancer with bleeding/hemorrhage or ulceration, mastitis, abscesses or trauma) [17,18]. As in the first scenario, the patient wears a surgical mask and follows the rules for social distancing in the waiting room. Radiological staff should wear an N95/FFP2 mask, goggles or face shield, gloves and cap. Ultrasound and mammographic machines must be covered by a plastic sheet and disinfected after the procedure with chloro-derivate solutions and the room should be open for correct air ventilation (>25 cycles/h) [9,16].
- **Third scenario – confirmed COVID-19 patient:** taking into account the highly contagious nature of SARS-CoV-2, breast imaging examinations or breast surgery should be postponed and rescheduled after two weeks from the first asymptomatic day unless it is urgent (Figure 2) [17,18]. In this case, the patient wears a surgical mask and stays isolated from other people. Radiological staff wears FFP3 masks, eye protection, impermeable full-length long-sleeved gown, gloves and cap. HCWs must pay maximal attention to dressing and undressing procedures, as suggested by Spallanzani Hospital [19]. Ultrasound and mammographic machines must be covered by a plastic sheet and disinfected after the procedure with chloro-derivate solutions and the room should be open for correct air ventilation (>25 cycles/h) [9,13,16].

### Methods at the department of breast surgery

All patients who received doubtful or positive assessments deserving of surgery at the Department of Diagnostic Senology, District 12, Caserta, were referred to our Department of Breast Surgery located at the Anastasia Guerriero Hospital of Marcianise (Caserta). Benign findings requiring surgery were delayed. In the case of a positive assessment, patients with confirmed breast cancer are discussed at the multidisciplinary team meeting (MDT) virtually attended by a breast radiologist, breast surgeon, oncologist and pathologist to avoid face-to-face contact.

All the necessary precautions must be taken before, during and after surgery and during patient transfer [20]. All the HCWs involved must be periodically trained about COVID-19 symptoms and prevention methods. Before the operation, all resources (devices, HCWs, intensive care beds) should be recorded to predetermine availability. This makes it possible to reserve stretchers, operating rooms and elevators for COVID-19-infected patients who need surgery. In the operating rooms, only the minimum number of HCWs should be admitted. They must wear all the necessary personal protective equipment, such as N95/FFP2 masks, sterile surgical gowns, disposable sterile gloves and caps, goggles or protective visors. Alcohol-based hand antiseptic is provided. Surgical staff wears FFP3 masks, eye protection, impermeable full-length long-sleeved gowns, gloves and caps. HCWs must pay maximal attention to dressing and undressing procedures, as suggested by Spallanzani Hospital [19].

### Postoperative care

To minimize the risk of readmission, patients were discharged as soon as they were clinically stable, usually one to three days after surgery. Postdischarge visits were reduced to the minimum required. If a patient developed COVID-19 related symptoms, a breast-care nurse or a trained radiographer, both identified as case managers, informed public health officers and family doctors to follow up on the infection's clinical course. They must also monitor all the established pathways after hospital discharge.

## Results

Following this organization model and according to the DTCP [11], from July 1st to October 31st, 2020, at the Department of Diagnostic Senology, District 12, Caserta, 994 exams were performed. In particular, 534 DBTs were performed as first-level screening evaluation, 126 DBTs as ambulatory exams, and 67 monolateral DBTs as oncologic follow-up or as second-level examinations. A total of 213 US examinations were also performed, including 121 ambulatory patients and 92 second-level screening exams (Table 1).

**Table 1.** Exams performed at the Department of Diagnostic Senology.

Exam	n	Type of exam/results
Digital breast tomosynthesis	727	534 as screening test
		126 as ambulatory exams
		67 monolateral as follow-up
Ultrasound	213	121 as ambulatory exams
		94 as second-level screening test
Fine-needle agoaspiration citology	28	C1: 1
		C2: 15
		C3: 2
		C4: 2
		C5: 8
Core needle biopsy	9	B2: 1
		B3: 1
		B5: 7
Tomobiopsy (vacuum-assisted breast biopsy with tomosynthesis)	17^†^	B2: 10
		B3: 3
		B5: 4

† 1 VABB was delayed and rescheduled after the end of the considered period.

In cases of suspicious findings at DBT or US, classified according to BI-RADS score [21], from B3 to B5, 28 FNAC, 9 CNB and 17 VABB procedures were performed (Table 1). One C1 was identified at FNAC that resulted in B2 at CNB; 15 C2, one of these also received VABB for a concurrent lesion, resulting in B2; two C3 resulted in B5 at CNB; two C4 resulted in B3 and B5, respectively, at CNB; and eight C5, 4 of these received CNB resulting in B5. Sixteen patients directly received VABB; of these, nine resulted in B2, three resulted in B3 and four resulted in B5. Sixteen breast cancers (BCs), 7 in women aged 50–69 years (screening range), 4 in women aged <50 years, 4 in women aged >70 years and one in a man aged 43 years old were identified (Table 2). Of these, 11 patients underwent surgery at the Department of Breast Surgery (two nipple-sparing mastectomy and 9 quadrantectomy), one patient started neoadjuvant chemotherapy and four patients were managed at other regional institutions.

**Table 2.** Pathological examinations with treatments for each diagnosis.

Age	Sex	FNAC	CNB	VABB	Diagnosis	Therapy
43	M	C4	B3		DCIS	Nipple-sparing mastectomy
28	F	C2			Fibroadenoma	
30	F	C2			Fibroadenoma	
42	F			B2	Fibrosis	
44	F			B5	DCIS	QUAD
46	F	C2			Cyst	
46	F			B5	DCIS	Unknown^†^
47	F	C1	B2		FBD	
48	F	C2			Cyst	
48	F			B5	Diffuse DCIS	Nipple-sparing mastectomy
49	F	C4	B5		Invasive ductal carcinoma with LCIS	QUAD
49	F			B3	Sclerosing adenosis with columnar cell hyperplasia	
50	F			B2	UDH	
50	F	C2			Fibroadenoma	
50	F	C5			Invasive ductal carcinoma	QUAD
50	F	C2			Cyst	
50	F	C2			FBD	
52	F			B2	Sclerosing adenosis	
52	F	C2			Fibroadenoma	
52	F	C5			Invasive ductal carcinoma	QUAD
54	F			B5	DCIS	QUAD
54	F	C2			Fibroadenoma	
54	F	C2		B2	Sclerosing adenosis	
54	F	C5			Invasive ductal carcinoma	Unknown^†^
55	F	C2			Seroma	
55	F	C5	B5		Invasive ductal carcinoma	Neoadjuvant chemotherapy
56	F			B2	UDH	
57	F	C2			Fibroadenoma	
59	F	C3	B5		Invasive ductal carcinoma	QUAD
60	F	C2			Fibroadenoma	
61	F	C2			Fibroadenoma	
61	F	C2			Atypical fibroadenoma	
62	F			B2	Apocrine metaplasia	
63	F			B2	Sclerosing adenosis	
63	F	C3	B5		Invasive ductal carcinoma	QUAD
64	F			B4	Apocrine metaplasia	
67	F			B3	UDH + ADH	
68	F			B2	UDH	
69	F			B3	ADH	
71	F	C5	B5		Multifocal ductal carcinoma	QUAD
74	F	C5	B5		Invasive ductal carcinoma	Unknown^†^
77	F			B2	UDH	
77	F	C5	B5		Invasive ductal carcinoma	QUAD
84	F	C5			Bilateral ductal carcinoma	Unknown^†^

† Patient received surgery elsewhere.

ADH: Atypical ductal hyperplasia; CNB: Core needle biopsy; F: Female; DCIS: Ductal carcinoma *in situ*; FBD: Fibrocystic breast disease; FNAC: Fine-needle agoaspiration cytology; LCIS: Lobular carcinoma *in situ*; M: Male; QUAD: Quadrantectomy; UDH: Usual ductal hyperplasia; VABB: Vacuum-assisted breast biopsy.

During the same period, at the Department of Breast Surgery, 54 surgical treatments were performed. Of these, 40 were performed for breast cancer patients (74.1%). Five women were identified as suspected COVID-19 patients and rescheduled. Two women, who received DBT, showed suspected SARS-CoV-2 infection symptoms two days after the exam; case managers informed their family doctors and they performed a RT-PCR test, with negative and positive results, respectively. For the patient affected, VABB was delayed and rescheduled beyond the period considered in this paper (at November, sclerosing adenosis, B2). Finally, no one from the medical staff was infected during this period.

## Discussion & conclusion

Due to the COVID-19 emergency, breast cancer units across Italy suffered a significant reduction in their clinical activity. Hospital resources were redistributed from elective and semielective procedures to COVID-19 patients in critical conditions [22], with a subsequent scarcity that could delay diagnostic evaluations and treatment for patients with BC. BC is the most frequent cancer in women of all ages, with more than 5000 early breast cancer (defined as carcinoma *in situ* or infiltrating cancer less than 1 cm) diagnoses every year [23]. The Italian national screening program improved the prognosis of patients with BC, reaching a survival rate of 87% at 5 years with a significant reduction of about 30% of tumors in advanced stages [24]. This reduction represents a relevant resource for our health system because it results in a lower use of adjuvant therapies, shorter surgery times, earlier return to work and a significant improvement in quality of life. The estimated doubling time of breast cancer ranges between 45 and 260 days [25]: this growth rate variability does not allow for the precise estimation of the impact on patients who could not undergo breast cancer imaging tests during the COVID-19 outbreak. The literature investigating the association between delay and prognosis in patients with BC has yielded conflicting results [26], but a short delay (e.g., 6–12 weeks) should not affect the overall outcome [8]. However, the periodic interruption of BC screening activities, following the spread of SARS-CoV-2 infection in the population, could have a considerable effect on the female population.

A recent study that compared the breast unit activity in the first half of 2020 to the same period in 2019 reported an increased number of referrals either for diagnostic exams in suspected BC patients (estimated around 28%) or for patients who received their first treatment for a BC diagnosis (estimated around 16%) [27]. Vanni *et al.* [28] estimated that 50% of the 11,000 cases will be identified with a delay of only 6 months, associated with a cancer stage progression. They report that 8125 BC diagnoses could be missed due to a screening interruption of three months [28]. This delayed diagnosis has several consequences, such as more invasive breast surgery or neoadjuvant or adjuvant therapy with a worse patient outcome and an increase in healthcare costs. Jacob L *et al.* [29] showed a significant reduction in new cancer diagnoses in Germany between March and May of 2020 compared with 2019. This trend was similar both in general and specialized practices and in almost all sex and age groups [29]. A study from a secondary care hospital in Italy revealed that the incidence of BC diagnoses decreased by 26% in 2020 [30] in comparison with 2018–2019, while Kaufman *et al.* showed a decrease of 51.8% in the United States [31].

In our experience, from July to October 2019, of a total of 1827 DBTs performed, 46 BC cases (2.5%) were identified. In particular, 36 BC were identified from 1736 screening exams (50–69 years) and 10 BC were identified from 88 ambulatory exams (four women aged 45–49 years and six >69 years). Referring to 2020, in the same time interval, of a total of 660 DBTs performed, 16 BC (2.4%) were identified. In particular, 7 BC were identified from 534 screening exams and 9 BC were identified form 126 ambulatory exams (five in women aged 45–49 years and four >69 years). A statistically significant difference (p < 0.01) was found in the data recorded among the two categories of patients (screening and ambulatory) in comparison with 2019 and 2020, such that the reduction of new diagnoses in the screening range reflect a massive decrease in the number of mammographies performed among 50–69 year-old asymptomatic women, in contrast with the increase in examinations for women in the other age ranges.

The reduction in the number of new BC diagnoses during the pandemic might also be caused by the patients' fear of contracting SARS-CoV-2 infection. In fact, women with a suspected breast lesion and those with diagnosed BC frequently refused surgery because of the risk of developing symptoms of COVID-19 [32], probably due to the belief that hospitals are infectious reservoirs [29]. The decreasing rate of healthcare utilization for nonurgent pathologies combined with the rapid overload of the healthcare system, due to the high number of patients infected with COVID-19, supported this trend [29] such that nonurgent care services were delayed or suspended. According to this scenario, triaging both patients' clinical status and urgent clinical cases, and awake and fast surgery are mandatory to optimize the allocation of the limited resources during higher peaks, increasing the number of treated patients and reducing hospitalizations as well as the risk of cross-infection [33].

It is also useful to underline that the spreading of COVID-19 infection can not only affect patients' decision-making process but may have also a psychological impact on HCWs. Ng *et al.* [34] found a high level of perceived risk, anxiety and fears in HCWs with a prevalence of anxiety (22.5%) and burnout (43.5%), but these values had not increased from pre-COVID-19 rates. Conversely, in Italy, Rossi *et al.* [35] found a high level of post-traumatic stress symptoms (49.4%), depression (24.7%), anxiety (19.8%), insomnia (8.3%), and high perceived stress (21.9%), especially in young women and frontline HCWs, while no difference was found in low-risk environments [33]. More investigations are needed since different perceptions of risk could be significantly influenced by effective public health interventions and the different number of confirmed patients with COVID-19 infection among countries (more in Italy and the United States than in China). Moreover, the Italian outbreak emerged mainly in the northern region, presenting a relevant difference in terms of cases and fatality rates across the country [33].

The management of patients and healthcare staff described here is an example of the practical use of national and international guidelines as applied to a Breast Cancer DTCP. The protocol may assist specialists in preventing COVID-19 infection and in optimizing resources for breast cancer diagnosis, especially in Southern Italy, where a low rate of improvement in prognosis has been recorded (84%), mostly related to a minor adherence rate to the organized screening program and a lack of knowledge about the importance of early diagnosis [10].

This study demonstrated efficacy in terms of continuity in the provision of an essential level of care in breast cancer screening and ambulatory settings. Since patients with cancer are forced to choose between seeking treatment and increasing the risk of contracting COVID-19, or postponing therapy and minimizing the risk of contracting the infection, more accessible information for the general population could be useful to clarify that the risk of contamination is relatively low when all effective protective measures are respected [36]. In our experience, this proposed model has resulted in a contraction of the waiting lists by a few weeks with a low rate of advanced breast cancer. The absence of medical and paramedical staff SARS-CoV-2 infection also shows the effectiveness of the infection prevention procedures adopted. Conscientiousness regarding how to improve safety at work, for both patients and clinicians, especially in such an extraordinary condition as a pandemic, is just as important as knowing the biological aspects of pathology. Since the SARS-CoV-2 outbreak seems far from over, this protocol may assist specialists and HCWs in preventing infection during this period, and in future similar conditions.

**Summary points**

- To prevent infection for both patients and healthcare workers during breast imaging procedures, patients were triaged using a telephone questionnaire and separated into three different clinical scenarios (non-COVID-19 patients, suspected COVID-19 patients, confirmed COVID-19 patients). Specific procedures were developed for each scenario.
- To prevent the spread of the infection, suspected or confirmed COVID-19 patients were postponed if not urgent, and rescheduled two weeks after first asymptomatic day.
- From July to October 2019, 16 breast cancers were identified in 7 in women aged 50–69 years (screening range) and 9 in the other age ranges; 11 patients underwent conservative treatments.
- From July to October 2020, 46 breast cancers were identified in 36 in women aged 50–69 years (screening range) and 10 in the other age ranges.
- Comparing 2019 and 2020, a statistically significant difference in the number of new breast cancer diagnoses was not found. However, the reduction of new diagnoses in the screening range in comparison with a substantially equal number of diagnoses among ambulatory patients reflects the massive decrease in the number of breast cancer screening mammographies performed, in contrast with the increase in examinations for symptomatic women in the other age ranges.
- A different level of perception of risk, anxiety, fears and other mental health disorders have been recorded among patients and HCWs, both in Italy and in other countries. More investigations are needed since this aspect of the pandemic could be significantly influenced by effective public health interventions and the differential outbreaks in the Italian regions and among other countries.
- This study demonstrates efficacy in terms of continuity in the provision of an essential level of care, and the absence of medical and paramedical staff SARS-CoV-2 infection is additional proof of the effectiveness of the infection prevention procedures adopted.
- Since the COVID-19 outbreak seems far from over, this protocol may assist specialists and health workers in preventing infection during this period and in future similar conditions.
